# Hidden risk factors and the mediating role of sleep in work-related musculoskeletal discomforts

**DOI:** 10.1186/s12891-024-07387-0

**Published:** 2024-04-02

**Authors:** Ebrahim Darvishi, Hiva Osmani, Abbas Aghaei, Emran Ali Moloud

**Affiliations:** 1 Department of Occupational Health Engineering, Faculty of Health, Kurdistan University of Medical Sciences, Sanandaj, Iran; 2 Environmental Health Research Center, Research Institute for Health Development, Kurdistan University of Medical Sciences, Sanandaj, Iran; 3 Student research committee, Kurdistan University of Medical Sciences, Sanandaj, Iran; 4 Department of Epidemiology and Biostatistics, Faculty of Medicine, Kurdistan University of Medical Sciences, Sanandaj, Iran

**Keywords:** Hidden risk factor, Shift work, Occupational stress, Sleep, Musculoskeletal discomforts

## Abstract

**Objective:**

Musculoskeletal discomforts (MSDs) are prevalent occupational health issues that are associated with a wide range of risk factors. This study aimed to investigate some of the occupational hidden risk factors and the mediating role of sleep in work-related musculoskeletal discomforts.

**Methods:**

In a cross-sectional study, the role of job stress and shift work as two hidden risk factors and sleep problems as the mediator in work-related musculoskeletal discomforts was investigated in 302 healthcare workers using the path analysis models. For this aim, healthcare workers’ Occupational Stress and musculoskeletal discomforts were evaluated using the Health and Safety Executive questionnaire and Cornell questionnaire, respectively. Moreover, the Pittsburgh Sleep Quality Index (PSQI) and the Insomnia Severity Index (ISI) were used to examine the sleep characteristics of participants. Shift work and job stress as predictor variables and sleep characteristics as mediating variables were analyzed.

**Results:**

The results showed that the path coefficients of job stress on indexes of quality sleep and insomnia severity were significant. Also, the path coefficient of shift work on quality sleep index was significant. In return, the path coefficients of shift work on the insomnia severity index were not significant. Additionally, there was a mutually significant association between indexes of quality sleep and the severity of insomnia and musculoskeletal discomforts. The direct effect coefficient of job stress on MSDs was significant, whereas the direct effect coefficient of shift work on MSDs was insignificant. This means that shift work alone does not significantly impact these disorders.

**Conclusion:**

It would seem that shift work and job stress as two occupational hidden risk factors can mediate sleep indexes and indirectly play a critical role in the incidence of musculoskeletal discomforts. Moreover, sleep disorders and musculoskeletal discomforts are mutually related and have a bidirectional relationship.

## Introduction

Musculoskeletal discomforts (MSDs) are prevalent occupational health issues affecting individuals in contemporary societies and among working populations [1, 2]. MSDs encompass a wide range of painful injuries and disorders that affect several anatomical tissues, including muscles, tendons, peripheral nerves, joints, vascular support structures, and the spinal column, in both the lower and upper limbs [3]. MSDs can affect workers in all sectors and occupations and can lead to high costs for enterprises and society. Based on the report from the European Agency for Safety and Health at Work, MSDs are the most prevalent work-related health issue in the European Union, accounting for over 50% of serious work-related diseases [4]. In Iran, MSDs have been reported as the main cause of disability and complaints in the workplace [5]. Moreover, many studies have shown that low back pain is the primary contributor to disability within the realm of non-communicable diseases, including in both industrialized and developing nations. In the year 2020, the global prevalence of low back pain exceeded 500 million cases [6]. According to projections, the prevalence of low back pain is expected to exceed 800 million individuals worldwide by the year 2050 [7].

MSDs have a progressive and multifaceted etiology, which is marked by a high level of diagnostic complexity [8]. There is widespread recognition that MSDs are strongly influenced by work-related factors. However, prior research has identified many risk factors, including personal, physical (biomechanical), and psycho-organizational aspects, associated with the development of MSDs [9]. Age, gender, body mass index, sleeping habits, systemic diseases and personality traits are the most important individual factors [3, 10–14]. There is no doubt that biodynamic risk factors such as poor posture at work, force exertion, repetition movement, prolonged sitting, vibration, and high workload play the main roles, and the influence exerted by these elements on the development of MSDs is well-recognized [15, 16]. The present emphasis on methods, policies, and programs to prevent MSDs mostly centers on physical variables, whereas psycho-organizational elements are often overlooked [17, 18]. Therefore, there are some hidden work-related risk factors such as shift work, job stress, and sleep problems that few studies have attempted to focus on. Shift workers may be at higher risk for developing MSDs compared to those who work regular daytime hours. Moreover, stress at work is a condition that can arise from various sources such as heavy workloads, long working hours, lack of control over tasks, inadequate job resources or support, poor relationships with colleagues or supervisors, and conflicts between work and personal life. Prolonged exposure to job stress can have adverse effects on human health [5].

Healthcare and hospital workers (HCWs) have a higher prevalence of MSDs, mainly affecting the back, neck, shoulder, and knee regions, three to four times higher than other occupational categories [19]. The high prevalence of MSDs in HCWs is often linked to physically demanding aspects of their job, such as lifting patients, prolonged periods of standing, and repetitive motions [20, 21]. Approximately one-third of sick days taken by HCWs are attributed to MSDs [22]. Sleep problems in HCWs are a recognized manifestation of job stress and shift work, a phenomenon that has been extensively reported among HCWs due to patient care 24 h a day [23]. Marvaldi et al. [24] reported that the prevalence of sleep disorders among nurses was 44.0% (95% CI, 24.6–64.5). Shift work can indeed have a significant impact on sleep, and many studies have reported high prevalence rates of sleep disorders among shift workers. The nature of shift work, with irregular or overnight hours, can disrupt the body’s natural sleep-wake cycle and lead to difficulties in getting adequate and restful sleep [5]. Moreover, job stress can be a significant factor contributing to sleep disorders. The demands and pressures of work can lead to increased levels of stress, anxiety, and worry, which in turn can disrupt sleep patterns. Common sleep disorders associated with job stress include insomnia, restless leg syndrome, and sleep apnea [25]. One study by Hamming found a weak association between sleep disorders and the development of MSDs [26]. Their findings report that general and occupational stress were reported to be significantly related to sleep disorders. However, another study found that in emergency HCWs, sleep disturbances are significantly associated with MSDs and occupational stress [27]. Moreover, according to studies, there is a bidirectional relationship between sleep problems and MSDs [28, 29], whereby sleep disturbances have been found to exacerbate symptoms of MSDs, while MSDs hinder the ability to achieve restful sleep. Some research has presented conflicting findings in this regard. Evaluation of the association between sleep problems and MSDs is a crucial matter for enhancing management strategies, preventing MSDs, reducing workforce impairment, increasing job satisfaction, increasing efficiency, and ultimately enhancing the quality of service provided to patients. Although it has been proven that awkward posture, force exertion, repetitive movements, manual material handling, and tasks are the main occupational risk factors in creating MSDs however, some other occupational risk factors such as shift work, job-related stress, and sleep issues may also indirectly contribute to the development of musculoskeletal disorders that have not been profoundly studied. Therefore, this study aimed to hypothesize that shift work and job stress are two hidden risk factors for MSDs, and in this relationship sleep problems are mediators. Moreover, the relationship between sleep problems, job stress, shift work, and musculoskeletal discomforts, and the bi-directional relationship between MSDs and sleep problems was investigated among healthcare workers.

## Method

### Study design and participants

In a cross-sectional study, the role of job stress and shift work as two hidden risk factors and sleep problems as the mediator in work-related musculoskeletal discomforts was investigated in 302 healthcare workers in two shift groups (rotation shift and morning shift) using the path analysis models. All participants were from three public hospitals in Kurdistan Province, Iran. The sample size was estimated based on the established methodology for determining sample sizes in correlation studies. Data collection begun in October and ended in November 2022. All participants at the workstation received questionnaires, accompanied by a detailed explanation of the questionnaire’s content and the need to provide accurate responses. 46.4% of participants were women, and 53.6% were men. The mean ± SD of age were 30.33 ± 6.99.

**Fig. 1:**
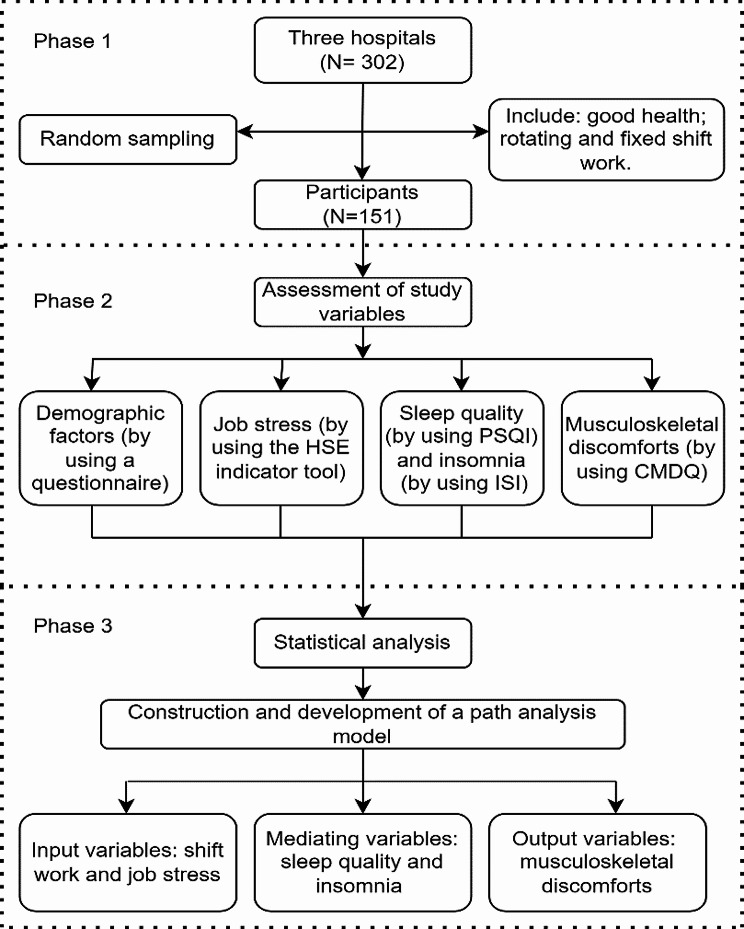
Flow diagram of the study design

This study has been approved by the Ethics Committee of the Kurdistan University of Medical Sciences. A demographic questionnaire was utilized to collect the participants’ characteristics, such as age, gender, job type, work experience, shift work, drug usage, involvement in sports activities, and history of physical and mental health conditions. Figure 1 represents the flow diagram of the study design.

### Sleep characteristics

The sleep characteristics of employees were assessed using two indexes of Pittsburgh Sleep Quality Index (PSQI) and the Insomnia Severity Index (ISI). PSQI has 19 items, which are scored on a 4-point Likert scale from 0 to 3. This questionnaire has 7 subscales, which are: subjective sleep quality, sleep latency, sleep duration, habitual sleep efficiency, sleep disturbances, use of sleeping medication, and daytime dysfunction. A score of 7 or higher indicates poor sleep quality [30]. The Insomnia Severity Index (ISI) is a brief self-report questionnaire used to assess the nature, severity, and impact of insomnia. It consists of 7 items that measure the perceived severity of both nighttime and daytime components of insomnia. The ISI is widely used in clinical practice and research to assess the existence and severity of insomnia. A total score exceeding 8 on the ISI suggests the presence of insomnia [31, 32].

### Occupational stress

Occupational stress was assessed using HSE’s indicator tool [33]. It comprises a total of 35 items and is organized into seven distinct subscales. These subscales are as follows: (1) demand; (2) control; (3) support from superiors; (4) support from colleagues; (5) communication; (6) role; and (7) changes. The questions are scored using a 5-point Likert scale, where the response options range from “never” (scored as 5) to “rarely” (scored as 4), “sometimes” (scored as 3), “often” (scored as 2), and “always” (scored as 1). A higher score on this questionnaire is indicative of lower and more suitable levels of Occupational stress, whereas a lower score suggests a higher degree of stress [34].

### Musculoskeletal discomforts

**Fig. 2:**
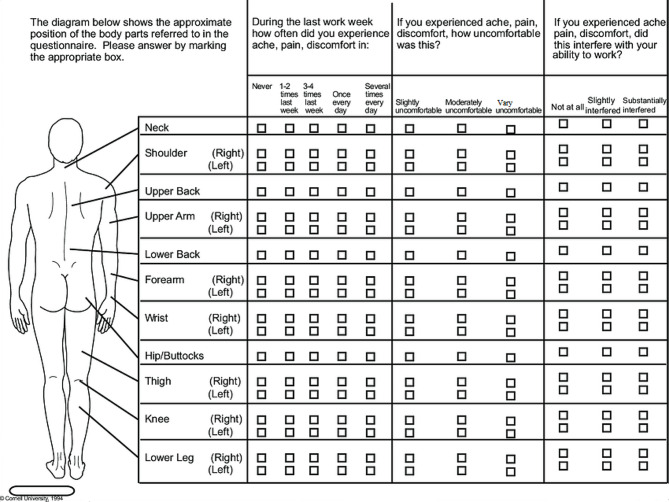
Structure of Cornell Musculoskeletal Discomfort Questionnaire (CMDQ) used for the present study

Cornell Musculoskeletal Discomfort Questionnaire (CMDQ) was used as an efficient tool to assess the severity of pain and discomfort experienced across 20 anatomical regions throughout the preceding workweek [35]. The CMDQ contains 57 questions covering various body regions, including the neck, shoulders, upper back, upper arm, lower back, forearm, wrist, hip, thigh, knee, lower leg, and foot. The participants were requested to evaluate the frequency of their discomfort using a scale that ranged from 0 (representing the absence of discomfort) to 4 (representing discomfort experienced daily). Furthermore, participants were instructed to assess the intensity of their discomfort using a numerical scale that ranged from 1 (representing mild discomfort) to 3 (representing significant discomfort). The extent to which the discomfort hindered work varied from 0 (no trouble) to 2 (considerable difficulty). Figure 2 illustrates the body map and the contents of the CMD Questionnaire.

### Variables and analysis models

Two hidden work-related risk factors of shift work and job stress were taken into account as input variables. Quality sleep index and insomnia severity index were two mediating variables and output variables were musculoskeletal discomforts. In this study, two path analysis models (PAM) were developed to explore the role of shift work, job stress, and sleep indexes on WMSD. Path Analysis Model (PAM) is a useful technique for examining the relationships between independent variables and a specific target (dependent) variable. In PAM, each path is assigned a coefficient, which represents the standardized partial regression coefficient. These coefficients range from − 1 to + 1. A higher coefficient indicates that the variable has a stronger impact on another variable. Essentially, PAM helps us understand the direct and indirect effects of different factors in a complex system. The fit goodness of the model can be determined using indices including the model X^2^ values, goodness-of-fit index (GFI), root mean square error of approximation (RMSEA), Normed fit index (NFI), and comparative fit index (CFI). All data analyses were performed using SPSS 24 and the SPSS Amos version 24.

## Results

The results showed that 46.4% of participants were women and 53.6% were men. The mean ± SD of age and work experience of participants were 30.33 ± 6.99 and 6.24 ± 4.90 years respectively. The mean ± SD of the body mass index of workers was 25.36 ± 3.48 kg/m^2^. Table 1 presents the prevalence rate of MSDs in the participants. As indicated, the highest level of discomfort has been reported in the neck, back, knees, and legs respectively.

**Table 1:** Prevalence rates of musculoskeletal disorders in the participants

Part of the body	Frequency score	Mean score	SD
Neck	2.40	13.51	23.23
Right Shoulder	1.70	7.76	17.84
Left Shoulder	1.33	6.77	18.82
Upper Back	1.68	7.34	15.54
Right Upper Arm	0.99	3.74	10.63
Left Upper Arm	0.72	2.36	8.520
Lower Back	2.35	11.06	21.56
Right Lower Arm	0.96	4.16	13.44
Left Lower Arm	0.77	3.76	13.36
Right Wrist	1.13	6.22	17.87
Left Wrist	0.95	4.60	13.20
Hip	1.03	5.72	17.49
Right Thigh	1.20	7.16	20.64
Left Thigh	1.17	5.83	18.60
Right Knee	1.67	10.01	22.50
Left Knee	1.81	9.36	21.98
Right Upper Feet	1.58	8.73	20.59
Left Lower Feet	1.44	8.19	21.14
Right Feet	1.81	9.98	22.34
Left Feet	1.74	10.18	23.28

**Fig. 3:**
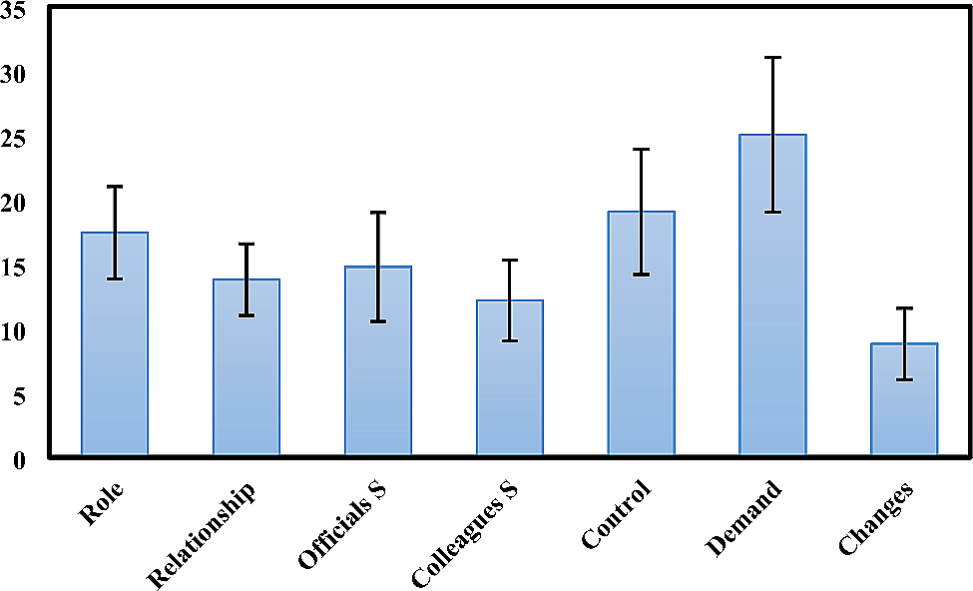
The average score of the seven sub-scales of job stress in the participants

Moreover, the mean ± SD of the total score of job stress in participants was estimated at 111.17 ± 16.68. Figure 3 illustrates the average score of the seven sub-scales of job stress in the participants.

The results of descriptive and analytical statistics of sleep variables based on Musculoskeletal discomforts have been provided in Table 2. The subscales of sleep latency and sleep duration have the highest rates. Table 2 shows that there is a significant relationship between indexes of sleep quality and insomnia severity with musculoskeletal discomforts.

**Table 2:** Descriptive and analytical statistics of sleep variables based on Musculoskeletal discomforts

Sleep Variables	Mean	SD	MSDS	
			Correlation Coefficient	*P*-value
Subjective sleep quality	1.17	0.69	0.302	<0.001
Sleep latency	1.23	0.91	0.282	<0.001
Sleep duration	1.25	1.00	0.064	0.442
Habitual sleep efficiency	0.44	0.83	0.272	<0.001
Sleep disturbances	1.17	0.57	0.223	0.006
Use of sleeping medication	0.40	0.79	0.179	0.029
Daytime dysfunction	0.89	0.94	0.236	0.004
**Total Sleep Quality Index**	6.52	3.46	0.341	<0.001
**Insomnia Severity Index**	8.29	4.77	-0.179	0.029

Table 3 provides the descriptive and analytical statistics of sleep variables and Musculoskeletal discomforts based on shift work. As can be seen, there is a significant difference between the two sub-scales of sleep latency and sleep duration and also the total indexes of sleep quality and insomnia severity in two shifts of fixed and rotating.

**Table 3:** Descriptive and analytical statistics of sleep variables and MSDs based on shift work

Sleep Specifications	Fixed Shift		Rotating Shift		*P*-value
	Mean	SD	Mean	SD	
Subjective sleep quality	0.91	0.53	1.28	0.73	0.472
Sleep latency	1.03	0.82	1.52	0.96	**0.041**
Sleep duration	1.31	0.70	1.27	1.07	**0.013**
Habitual sleep efficiency	0.16	0.45	0.53	0.92	0.230
Sleep disturbances	1.06	0.50	1.22	0.60	0.203
Use of sleeping medication	0.28	0.77	0.43	0.83	0.085
Daytime dysfunction	0. 63	0.71	1.00	0.99	0.135
**Total sleep quality index**	5.38	2.27	7.07	3.72	**0.007**
**Insomnia Severity Index**	6.63	4.09	8.81	4.93	**0.039**
**Job Stress**	48.5	9.41	26.33	7.45	**<0.001**
**Musculoskeletal discomforts**	116.34	257.63	183.98	272.49	0.083

**Fig. 4:**
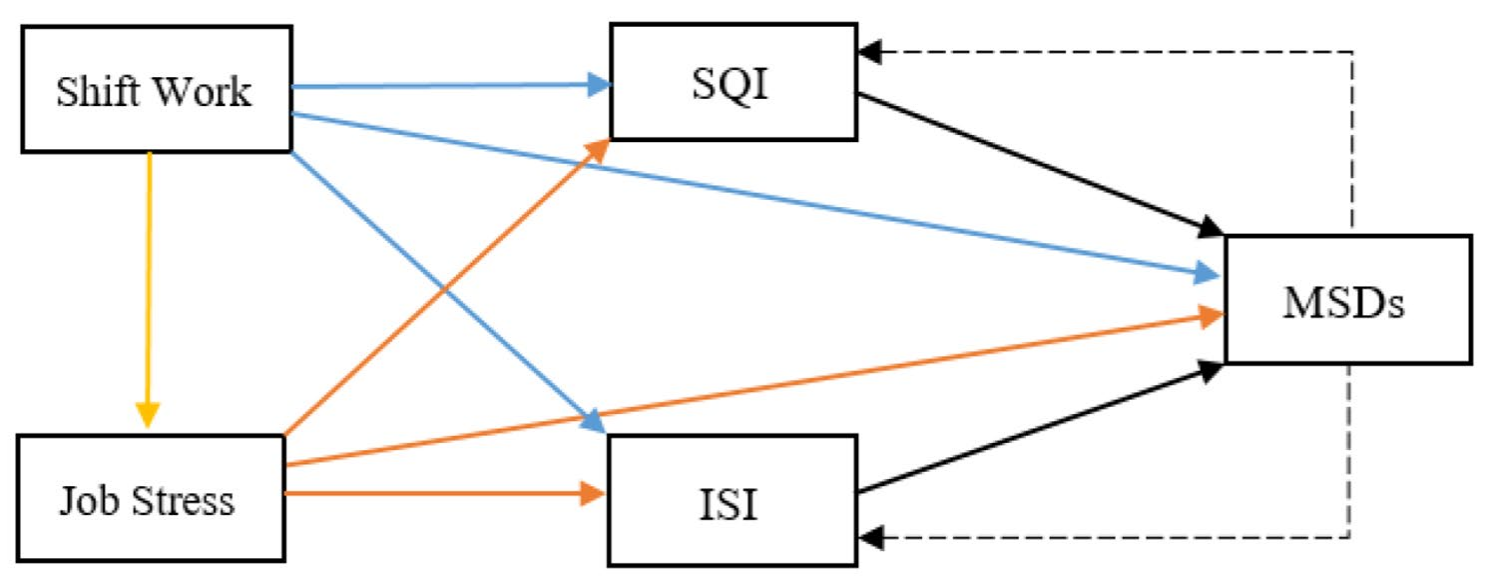
The model of explaining the direct and indirect impact of job stress and shift work on MSDs and the mediating role of sleep indexes (SQI: The Sleep Quality Index, ISI: Insomnia Severity Index)

Figure 4 illustrates the assumed model explaining the path analysis of job stress and shift work on musculoskeletal discomforts directly and indirectly by mediating sleep indices.

The results of the pathway analysis model and direct and indirect path coefficients of job stress and shift work on MSDs and the significance level of each path have been shown in Table 4. As shown, the path coefficients of job stress on indexes of quality sleep and insomnia severity were significant. Also, the path coefficient of shift work on quality sleep index was significant. In return, the path coefficients of shift work on the insomnia severity index were not significant. Additionally, Table 4 indicates a mutually significant association between indexes of quality sleep and severity of insomnia and musculoskeletal discomfort.

**Table 4:** The results of the pathway analysis model and direct and indirect path coefficients of job stress and shift work on musculoskeletal disorders

Path			Unstandardized path coefficient	Standardized path coefficient	SE	CR	*P*-value
From		To					
Shift Work	⇢	SQI	06.70	0.54	0.017	04.82	<0.001
Shift Work	⇢	Job Stress	0.328	0.18	0.067	1.124	0.043
Shift Work	⇢	ISI	04.08	0.08	0.080	1.101	0.152
Shift Work	⇢	MSDs	0.204	0.01	0.153	0.133	0.227
Job Stress	⇢	SQI	-0.064	-0.30	0.017	-3.772	<0.001
Job Stress	⇢	ISI	-0.110	-0.34	0.023	-4.840	<0.001
Job Stress	⇢	MSDs	-0.504	-0.15	0.458	-0.664	0.027
ISI	⇢	MSDs	7.628	0.35	1.565	4.531	<0.001
SQI	⇢	MSDs	8.143	0.46	2.479	2.982	<0.001
MSDs	⇢	SQI	0.008	0.26	0.002	3.461	<0.001
MSDs	⇢	ISI	0.015	0.32	0.003	4.797	<0.001

SQI: The Sleep Quality Index, ISI: Insomnia Severity Index, MSDs: Musculoskeletal discomforts

Table 5 reveals the fit indices of the developed pathway analysis model. As Table 5 shows, all the values were nearly acceptable.

**Table 5:** Fit indices of the developed pathway analysis model

Model fit index	Values		Acceptable level
	Indirect Model	Direct Model	
**Chi-square/df**	3	2.84	<3
**RMSEA**	0.079	0.077	<0.08
**IFI**	0.931	0.917	> 0.9
**TLI**	0.907	0.900	> 0.9
**GFI**	0.941	0.918	> 0.9
**CFI**	0.910	0.900	> 0.9
**NFI**	0.905	0.805	> 0.9

Note: RMSEA = Root Mean Square Error of Approximation; IFI = Incremental fit Index; NFI = Normed Fit Index; GFI = Goodness-Of-fit Index; CFI = Comparative fit Index; TLI = Tucker-Lewis’s index

## Discussion

Previous research has highlighted that healthcare and hospital workers, particularly nurses, experience a relatively high incidence of work-related musculoskeletal discomforts (MSDs), sleep disturbances, and stress [26]. These issues are often attributed to factors such as awkward postures, repetitive movements, manual material handling, physically demanding work environments, and mental workloads. Given the demanding nature of their jobs, it is crucial to explore additional risk factors. Therefore, few studies have specifically examined the combined impact of shift work, work-related stress, and sleep disorders on MSDs among healthcare professionals. Shift work disrupts natural sleep patterns, leading to misalignment between work hours and the body’s internal clock. This misalignment can contribute to health issues. Additionally, stress—both on and off the job—plays a significant role in the well-being of healthcare workers. Therefore, understanding the interplay between these factors is essential for developing effective preventive strategies. In the present study, the relationship between sleep problems, job stress, shift work, and MSDs with the moderating effect of sleep problems, and the bi-directional relationship between MSDs and sleep problems was investigated among healthcare workers. Finally, the causal paths were assumed for shift work and job stress as potential and hidden risk factors and sleep indexes as mediating variables in the creation of MSDs. We found that sleep quality (and its 6 subscales) and insomnia were significantly associated with MSDs. A meta-analysis study [36], based on 17 cross-sectional and cohort studies, found that chronic low back pain is linked to various sleep-related issues. These include heightened sleep disorders, decreased sleep duration and quality, prolonged time to fall asleep, impaired daytime functioning, and increased dissatisfaction and distress related to sleep. Also, a cross-sectional study among 450 patients reported that insomnia is associated with musculoskeletal pain [37]. The precise mechanism underlying the relationship between sleep and musculoskeletal discomforts remains unclear. Research conducted on adult populations has demonstrated that sleep deprivation decreases pain tolerance and results in increased pain sensitivity [38]. In a previous review, Finan et al. investigated the main central pathways that are involved in the regulation of pain and sleep [39]. The authors highlighted changes in the opioidergic and monoaminergic pathways linked to chronic pain disorders, which could explain disruptions in wakefulness and sleep cycles.

The findings of this study also indicated a significant difference in indexes of sleep quality and insomnia severity in two shift working groups. However, there is extensive evidence of sleep disturbance caused by shift work in workers who work unusual hours [40]. But even though there is no significant difference in MSDs in the two shift working groups, it was considered that the prevalence rate of MSDs was higher in rotating shift working groups.

As assumed, the results based on the path model indicated that job stress has a weak direct effect on MSDs, whereas it has a potential effect on MSDs through the mediating role of sleep quality and insomnia. A study among HCWs found that job stress is strongly associated with MSDs [26]. Li et al. [41], Constructed a Bayesian model demonstrating the direct effect of job stress on MSDs. Evidence shows that job stress is a hidden risk factor for developing MSDs [42]. Furthermore, psychological stress can lead to the ongoing release of catechol amines and cortisol, potentially impeding the process of musculoskeletal healing [43]. Experiencing job stress can have psychological, physiological, and behavioral effects on a person. This can cause changes in body chemistry that increase the chances of developing MSDs [44, 45].

The pathway analysis model also depicted that shift work does not affect MSDs directly and sleep indexes play a mediating role in this relationship. Even though shift work has been associated with an increased risk of developing MSDs due to factors such as prolonged standing or sitting, repetitive tasks, and constant physical exertion, however, research suggests the disruption of circadian rhythms caused by irregular sleep patterns and reduced time for rest and recovery can also contribute to musculoskeletal issues [40].

Based on the path model, MSDs have a bi-directional association with insomnia that is stronger than sleep quality. Consistent with this finding, there was a prospective and bi-directional association between poor sleep quality and chronic low back pain in school teachers [46]. Conversely, a previous study showed that previous lower back pain was a factor in causing sleep disruption [47]. Our developed conceptual model demonstrated that insomnia, sleep quality, and job stress were all predictive factors for MSDs. Therefore, based on the results, strategies and interventions should be developed to address these risk factors and mitigate the occurrence of MSDs in the healthcare setting. Job stress reduction programs, such as stress management techniques and workload management, can be implemented to minimize the impact of stress on healthcare workers’ musculoskeletal health. Shift work schedules should be designed to minimize the disruption of circadian rhythms and ensure adequate rest and recovery periods for healthcare workers.

### Limitation

This is a cross-sectional study, and these types of studies have several limitations. Firstly, because it is a one-time measurement, it is difficult to derive causal relationships from the cross-sectional analysis. Secondly, we used a self-reported questionnaire, and the obtained data cannot reveal an in-depth relationship between job stress, sleep problems, and MSDs. Additionally, the Pittsburgh Sleep Quality Index (PSQI) was employed to evaluate sleep quality, which is a subjective approach. For a more accurate assessment of sleep quality, we recommend using instrumental methods such as polysomnography. Thirdly, this study does not take into account various job-related factors and intra-individual changes that may have an impact on the development of MSDs among healthcare workers.

## Conclusions

The findings of this study strongly support this postulate that occupational stress and rotating work shifts can be potential and hidden risk factors for MSDs that have been identified as predictors or correlates of poor sleep and only secondarily can determine and equally cause MSDs. Therefore, sleep problems can cause MSDs, which MSDs also in turn produce sleep disorders. In other words, MSDs can be indirectly developed by work stress and shift work, whereas sleep disorders are only or mainly directly caused by shift work and stress and also can result directly from MSDs. These findings have implications for improving the overall health, safety, and well-being of healthcare workers.

## Data Availability

The datasets generated and/or analyzed during the current study are not publicly available due to limitations of ethical approval involving the patient data and anonymity but are available from the corresponding author on reasonable request.

## Abbreviations

MSDs: Musculoskeletal discomforts
PSQI: Sleep Quality Index, ISI: Insomnia Severity Index
